# Neurodegeneration in SCA14 is associated with increased PKCγ kinase activity, mislocalization and aggregation

**DOI:** 10.1186/s40478-018-0600-7

**Published:** 2018-09-24

**Authors:** Maggie M. K. Wong, Stephanie D. Hoekstra, Jane Vowles, Lauren M. Watson, Geraint Fuller, Andrea H. Németh, Sally A. Cowley, Olaf Ansorge, Kevin Talbot, Esther B. E. Becker

**Affiliations:** 1 Department of Physiology, Anatomy and Genetics, University of Oxford, Sherrington Road, Oxford, OX1 3PT UK; 2 Sir William Dunn School of Pathology, University of Oxford, South Parks Road, Oxford, OX1 3RE UK; 3 Gloucestershire Hospitals, NHS Foundation Trust, Cheltenham General Hospital, Sandford Road, Cheltenham, GL53 7AN UK; 4 Nuffield Department of Clinical Neurosciences, University of Oxford, Level 6, West Wing, John Radcliffe Hospital, Oxford, OX3 9DU UK; 5 Oxford Centre for Genomic Medicine, ACE Building, Oxford University Hospitals NHS Trust, Nuffield Orthopaedic Centre, Windmill Road, Oxford, OX3 7HE UK

**Keywords:** Ataxia, Stem cells, Purkinje cells, Neurodegeneration, Cerebellum, Protein kinase C gamma

## Abstract

Spinocerebellar ataxia type 14 (SCA14) is a subtype of the autosomal dominant cerebellar ataxias that is characterized by slowly progressive cerebellar dysfunction and neurodegeneration. SCA14 is caused by mutations in the *PRKCG* gene, encoding protein kinase C gamma (PKCγ). Despite the identification of 40 distinct disease-causing mutations in *PRKCG*, the pathological mechanisms underlying SCA14 remain poorly understood. Here we report the molecular neuropathology of SCA14 in post-mortem cerebellum and in human patient-derived induced pluripotent stem cells (iPSCs) carrying two distinct SCA14 mutations in the C1 domain of PKCγ, H36R and H101Q. We show that endogenous expression of these mutations results in the cytoplasmic mislocalization and aggregation of PKCγ in both patient iPSCs and cerebellum. PKCγ aggregates were not efficiently targeted for degradation. Moreover, mutant PKCγ was found to be hyper-activated, resulting in increased substrate phosphorylation. Together, our findings demonstrate that a combination of both, loss-of-function and gain-of-function mechanisms are likely to underlie the pathogenesis of SCA14, caused by mutations in the C1 domain of PKCγ. Importantly, SCA14 patient iPSCs were found to accurately recapitulate pathological features observed in post-mortem SCA14 cerebellum, underscoring their potential as relevant disease models and their promise as future drug discovery tools.

**Electronic supplementary material:**

The online version of this article (10.1186/s40478-018-0600-7) contains supplementary material, which is available to authorized users.

## Introduction

Spinocerebellar ataxia type 14 (SCA14) (OMIM 605361) most commonly represents with slowly progressive, relatively pure cerebellar ataxia characterized by gait disturbance, incoordination, mild dysarthria and nystagmus, with complex phenotypes such as myoclonus described in over a third of cases [9, 12]. Brain MRI in SCA14 patients shows mild to severe cerebellar atrophy [9, 12], and loss of Purkinje cells has been described at post-mortem [7].

**Fig. 1:**
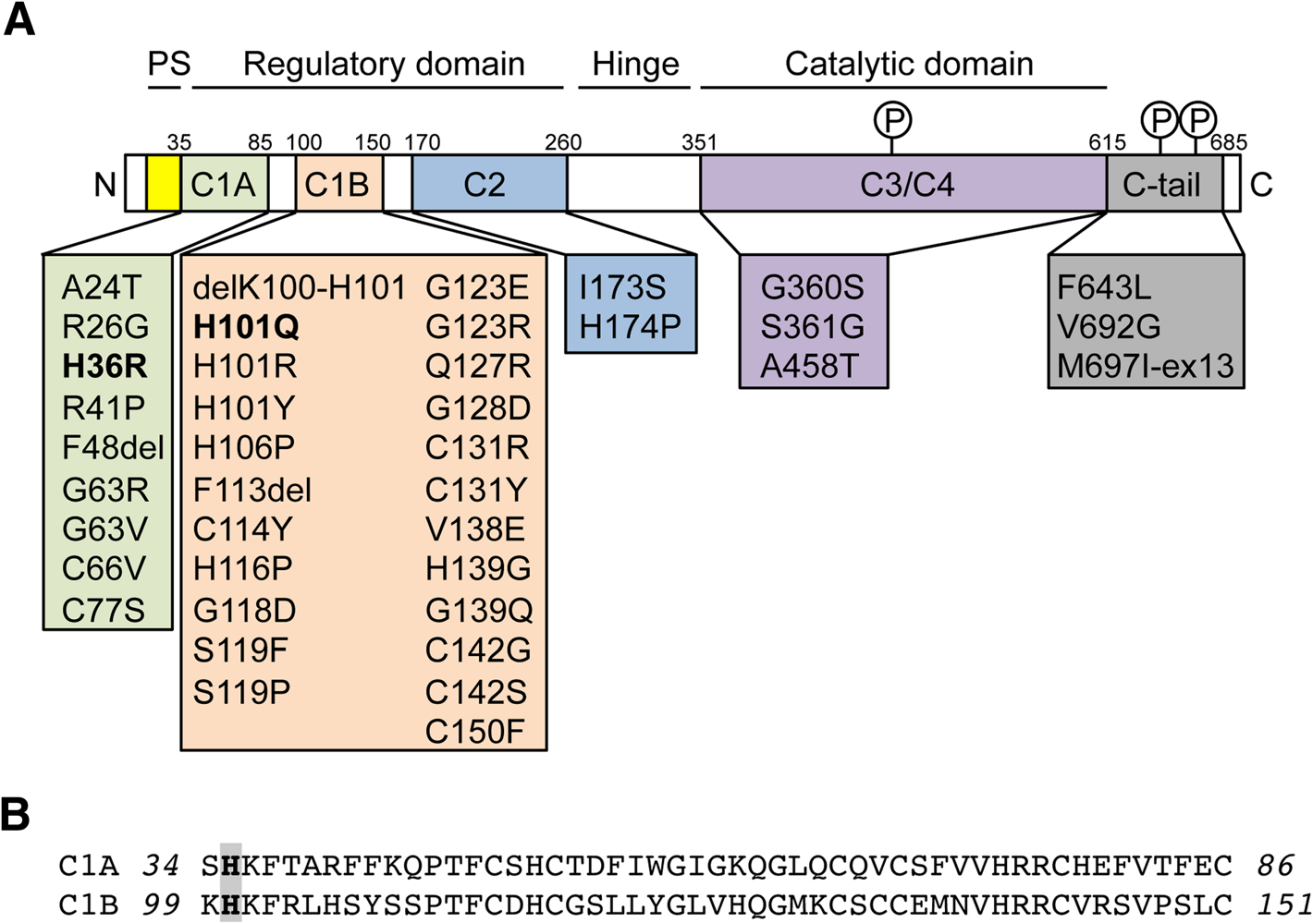
PKCγ mutations. **a** Domain structure of PKCγ. The localization of all reported SCA14 mutations is indicated. The two mutations investigated in this study (H36R, H101Q) are highlighted in bold. PKCγ is phosphorylated (P) at three conserved sites: at T514 in the catalytic domain and at T655 and T674 in the C-tail. PS: pseudosubstrate. **b** Sequence alignment of the two cysteine-rich subdomains C1A and C1B. The histidine residues at positions 36 and 101 (highlighted in bold) are located at equivalent positions within the two subdomains

SCA14 is caused by mutations in the *PRKCG* gene encoding the conventional protein kinase C gamma (PKCγ), which is particularly abundant in the Purkinje cells of the cerebellum [26]. To date, 40 mutations have been reported to cause SCA14 (Fig. 1a). Most of these mutations cluster in the regulatory C1 and C2 domains of PKCγ that respond to second messengers and control the activation and membrane translocation of PKCγ. Binding of calcium to the C2 domain initiates the activation of PKCγ and induces the rapid translocation of PKCγ from the cytoplasm to the plasma membrane, where it interacts with phospholipids. PKCγ is further allosterically activated by the binding of diacylglycerol (DAG) to the C1 domain, resulting in the release of a pseudo-inhibitory substrate that occupies the catalytic domain, and an open and active confirmation of PKCγ that allows phosphorylation of target substrates [2, 8].

The C1 domain is composed of two structurally and functionally similar cysteine-rich subdomains, C1A and C1B, of which the latter is preferentially affected by SCA14 mutations (Fig. 1a). Despite the wealth of mutations identified in PKCγ, the pathologic mechanisms underlying SCA14 remain unclear. Homozygous *Prkcg* knockout animals display only mild ataxia and show no loss of Purkinje cells [10, 22]. Therefore, the SCA14 phenotype is thought to result from a gain-of-function mechanism rather than haploinsufficiency. However, overexpression studies in cell lines and animals have yielded conflicting cellular disease mechanisms including increased kinase function [1, 43], impaired kinase function [42], protein aggregation [36] and impaired ubiquitin proteasome degradation [38], as well as aggregation-independent pathologies [37]. Thus, there is a need for authentic SCA14 models to better understand the underlying disease mechanisms.

Here, we have investigated the consequences of physiological expression of two SCA14 mutations in the C1 domain, H36R and H101Q, in both patient-derived induced pluripotent stem cells (iPSCs) and in SCA14 (H101Q) post-mortem cerebellum. We demonstrate that SCA14 patient iPSCs, in which PKCγ is expressed at levels more likely to be relevant to normal physiology compared with previous in vitro models, recapitulate pathological features observed in post-mortem SCA14 cerebellum. We found that the SCA14 mutations result in a decrease of PKCγ at the plasma membrane upon activation, but increased PKCγ aggregates in the cytoplasm of both patient iPSCs and Purkinje cells. We also observed lysosomal and autophagy impairment in SCA14 iPSCs and cerebellar tissue. PKCγ phosphorylation and downstream signaling were increased in the SCA14 iPSCs and cerebellum. Together, our findings suggest that SCA14 pathology is likely to be caused by the combination of a loss-of-function of PKCγ at the plasma membrane and a gain-of-function of hyper-activated and mislocalized PKCγ.

## Materials and methods

### Generation and maintenance of iPSC lines

iPSC lines were derived from four SCA14 patients, two carrying an H36R and two carrying the H101Q mutation (Additional file 1: Figure S2). Reprogramming of donor fibroblasts to iPSCs was performed as described in the Additional file 1, and full characterization is provided in Additional file 1: Figure S2. iPSC lines from two age- and sex-matched healthy donors were used as controls (Additional file 1: Figure S2) and have been fully described elsewhere [16, 18]. iPSCs were maintained in feeder-free conditions on hESC-qualified Matrigel (Corning), in supplemented mTeSR (Stem Cell Technologies). Cells were passaged 1:3 every 4–5 days, using 0.5 mM EDTA (Invitrogen) [5]. For inhibitor experiments, iPSCs were treated with 400 nM phorbol-12-myristate-13-acetate (PMA; R&D Systems) or 200 nM phorbol-12, 13-dibutyrate (PDBu) in PBS before harvesting.

### Quantitative real-time PCR

Total RNA from iPSCs and cerebellar tissue was prepared using the RNeasy Mini Kit (Qiagen). RNA from human fetal cerebellar tissue was purchased (AMS Biotechnology (Europe) Ltd., Abingdon, UK). RNA was reverse transcribed to cDNA using the High-Capacity RNA-to-cDNA Kit (Applied Biosystems). Quantitative real-time PCR was performed using the Fast SYBR Green Master Mix (Applied Biosystems) on a StepOne Plus qPCR machine (Applied Biosystems). The relative *PRKCG* levels were quantified and normalized against the housekeeping gene β-actin with reference to a negative control, using standard DDCt techniques. Primers are listed in Additional file 1: Table S1.

### Biochemical assays

Frozen tissue of human cerebellum was sampled from the inferior aspect, immediately lateral to the cerebellar tonsils (that is, adjacent to the areas with relative preservation of Purkinje cells on histology). Sampling sites were consistent between SCA14 and control cerebellum.

For the preparation of protein extracts, iPSCs were washed once with PBS and then lysed in cold Pierce® RIPA buffer [25 mM Tris-HCl pH = 7.6, 150 mM NaCl, 1% NP-40, 1% sodium deoxycholate, 0.1% sodium dodecyl sulphate (SDS)] (Thermo Fisher Scientific), supplemented with Complete Protease Inhibitor Cocktail (Roche) and PhosSTOP Phosphatase Inhibitor Cocktail (Roche). Protein lysates were incubated on ice for 10 min and subsequently centrifuged at 14,000 *g* for 20 min at 4 °C. Snap-frozen human cerebellar tissue was homogenized in cold RIPA buffer, followed by 30-s sonication. The cerebellar lysate was incubated on ice for 10 min before centrifugation at 14,000 *g* for 30 min at 4 °C. 50 μg of protein extracts were analyzed by SDS-PAGE and immunoblotting.

For the preparation of (in)soluble fractions, snap-frozen cerebellar tissue was homogenized in cold lysis buffer [1% Triton X-100, 20 mM Tris, pH = 7.5, 5 mM ethylene glycol-bis(β-aminoethyl ether)-N,N,N′,N′-tetraacetic acid (EGTA), 150 mM NaCl, Complete Protease Inhibitor Cocktail, PhosSTOP Phosphatase Inhibitor Cocktail], followed by a 10-min incubation on ice and centrifugation at 14,000 *g* for 30 min at 4 °C. The supernatant was collected as Triton-soluble fraction. The pellet was re-suspended in cold Pierce® RIPA buffer (Triton-insoluble fraction) and sonicated for 10–20 s. Equal volumes of soluble and insoluble fractions were loaded for SDS-PAGE and analyzed by immunoblotting.

A list of primary and secondary antibodies can be found in Additional file 1: Table S2. Antibody binding was detected by enhanced chemoluminescence (ECL, GE Healthcare). The intensity of bands was quantified using ImageJ software (NIH). Data were normalized to Actin levels and respective control bands and analyzed using GraphPad Prism 7 (GraphPad Software, Inc.). All data are represented as the mean of three independent experiments ±SEM. Statistical significance was assessed by ANOVA with Bonferroni’s post-hoc test, with *p* < 0.05 considered statistically significant.

### Immunostaining

iPSCs were fixed in 4% paraformaldehyde at room temperature for 20 min or in ice-cold methanol at -20 °C for 15 min. Fixed cells were washed three times with PBS for 5 min and subjected to immunostaining as previously described [44]. A list of primary and secondary antibodies can be found in Additional file 1: Table S2. Images were analysed using ImageJ. The size of aggregates was measured using the ImageJ Cell Counter plugin. The area of signals was measured using Threshold and Area Measurement. The co-localization of labelled proteins was quantified using Just Another Colocalization Plugin (JACoP) [6].

Immunohistochemistry of human cerebellar sections was performed as follows. 5-μm sections were cut from formalin-fixed paraffin-embedded blocks from the vermis, paravermis and lateral neocerebellum, including dentate nucleus. The index case was matched to two control cases. All three cases were assessed for PKCγ reactivity outside the cerebellum and screened for age-related neurodegenerative pathology. De-identified sections were de-waxed through xylene, and rehydrated through decreasing concentrations of alcohol before being pre-treated for 30 min in 10% concentrated (30%) H_2_O_2_ and distilled water to block endogenous peroxidase. Heat-induced epitope-retrieval was performed using autoclave boiling at 121 °C for 10 min. Sections were then rinsed with Tris-buffered saline and blocked with normal goat serum (1:10 in TBS-T) for 30 min. Primary antibodies (Additional file 1: Table S2) were incubated overnight at 4 °C and visualized using the Dako Envision+ kit and HRP-DAB signal (Agilent). Positive and negative controls were used for each antibody. No staining was seen when the primary antibody was omitted. Sections were viewed and photographed with an Olympus BX43 microscope and Olympus cellSense software.

## Results

### Cerebellar pathology in SCA14

Given the multitude of, often conflicting, phenotypes reported in the literature that might be caused by mutations in PKCγ in heterologous models, we set out to investigate the pathological changes in SCA14 in patient-derived cells and post-mortem cerebellum. We focused on two different SCA14 mutations, H36R and H101Q. Both of these mutations are located at equivalent positions in the C1A and C1B subdomains of the regulatory domain of PKCγ (Fig. 1) [32] and are implicated in zinc coordination and phorbol ester binding [11].

**Fig. 2:**
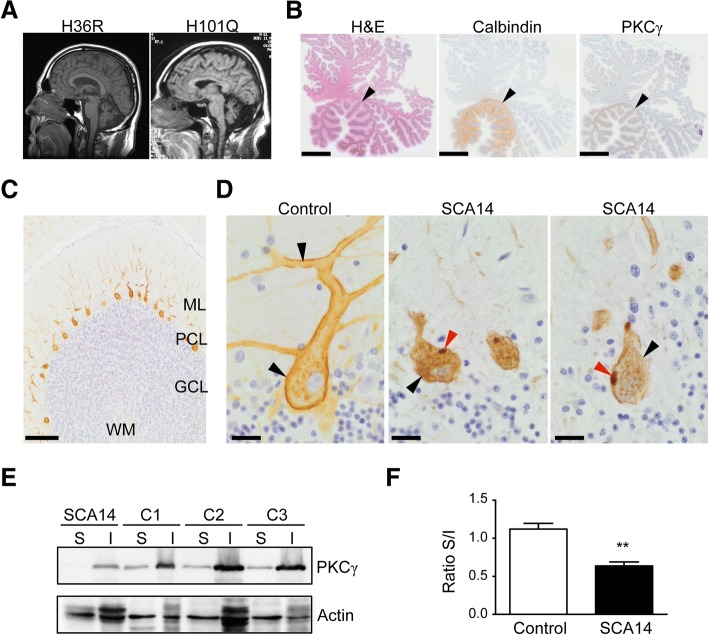
Cerebellar pathology in SCA14. **a** Brain MRI imaging of SCA14 patients carrying the H36R and H101Q mutations, respectively, shows marked cerebellar atrophy. **b** Neurodegeneration of SCA14 cerebellum. There is severe loss of Purkinje cells from the lateral neocerebellum. Purkinje cells in the tonsil (and flocconodular lobe) are relatively preserved (black arrowheads). Brain sections were stained with hematoxylin and eosin (H&E) (left panel), and with antibodies against Calbindin-28 k (centre) and PKCγ (right panel). Scale bar: 5 mm. **c** Normal PKCγ pattern from an age-matched control cerebellum. ML: molecular layer, PCL: Purkinje cell layer, GCL: granule cell layer, WM: white matter. Scale bar: 200 μm. **d** PKCγ staining of control (left) and SCA14 cerebellum (centre and right panels). In control cerebellum, PKCγ showed distinct expression at the plasma membrane, both around the soma and primary and secondary dendrites (black arrowheads), with minor granular staining in the perinuclear cytoplasm. In SCA14 cerebellum, homogeneous circumferential plasmalemma localization was lost (black arrowheads) and large cytoplasmic PKCγ aggregates were found, some apparently still linked to fragments of plasma membrane (red arrowheads). Scale bar: 20 μm. **e**, **f** Enrichment of mutant PKCγ in the Triton-X-100-insoluble fraction in SCA14 cerebellum compared to controls. Cerebellar tissue lysates were separated into Triton-X-100-soluble (S) and -insoluble (I) fractions. Equal volumes of soluble and insoluble fractions were loaded for SDS-PAGE and analyzed by immunoblotting for PKCγ. (**e**). Actin: loading control. The intensity of the bands was quantified and the level of PKCγ was normalized against the loading control. The ratio of normalized PKCγ present in the soluble versus insoluble fractions (Ratio S/I) is shown (*n* = 3, **p < 0.01, unpaired students’ t-test). (**f**). S: Triton-X-100-soluble fraction, I: Triton-X-100-insoluble fraction

Affected individuals had slowly progressive adult onset ataxia typical of SCA14, with moderate gait ataxia, mild dysarthria, titubation and relatively mild nystagmus. The H101Q family had pure ataxia, without additional features described in other pedigrees such as myoclonus and seizures. MRI showed moderate to severe generalized atrophy of the cerebellum (Fig. 2a). One individual from the SCA14 H101Q family underwent autopsy when he died of ‘natural causes’ at the age of 90 years (Additional file 1: Figure S1). Brain tissue was examined according to standard protocols for neurodegenerative disease, which included screening for Alzheimer disease, Lewy body disease and TDP-43 proteinopathy. We found Braak II/III neurofibrillary Alzheimer type pathology and mild cerebrovascular disease. No Lewy body or TDP-43 proteinopathy was identified. We used a sequestosome1/p62 antibody as a highly sensitive screening tool for generic protein aggregates. We did not find any neuropathology that could not be explained by Alzheimer-related changes.

To identify Purkinje cells, tissue sections were immunolabelled with an antibody against the calcium-binding protein Calbindin D-28 k. We observed severe loss (estimated to be 80%) of Purkinje cells in all lobules of the neocerebellum, associated with Bergmann gliosis. However, Purkinje cells in the cerebellar tonsils and adjacent flocculonodular lobe were relatively preserved (Fig. 2b). Neurons of the deep cerebellar nuclei, pons and inferior olive were not obviously depleted. Thus, we conclude that SCA14 seems to be a pure Purkinje cell neuronopathy, predominantly affecting the lateral parts of the cerebellar hemispheres (neocerebellum). This is consistent with the highly restricted expression pattern of PKCγ in human control cerebellum (Fig. 2c). No other cerebellar cell type expressed PKCγ. The remaining Purkinje cells displayed variable degrees of dendritic and somatic atrophy compared to control tissue (Fig. 2d). In age-matched control autopsy material, PKCγ was localized to the plasma membrane and cytoplasmic puncta in the soma and primary dendrite of Purkinje cells (Fig. 2d). This staining pattern is consistent with the localization of PKCγ in rodent Purkinje cells [26, 39]. In contrast, PKCγ staining at the plasma membrane was lost in SCA14 Purkinje cells and associated with large cytoplasmic aggregates in the soma, sometimes preserving a link to the plasma membrane (Fig. 2d). Loss of PKCγ staining was particularly pronounced in the dendrites. PKCγ aggregates were unique to Purkinje cells. Compared to Purkinje cells, only minimal expression of PKCγ is seen in any other part of the adult human brain. In our hands, the only extracerebellar region with faint expression in age-matched controls corresponded to the CA1-CA4 sectors of the hippocampus. However, unlike in the cerebellum, staining revealed only diffuse neuropil positivity, and no distinct membrane, soma, dendrite or axonal neuronal expression (data not shown). The SCA14 index case showed no aggregates or other morphological PKCγ abnormalities in the hippocampal formation compared with controls. We conclude from our immunohistochemical studies that cytoplasmic and membrane expression of PKCγ in adult cerebellar Purkinje cells is several orders of magnitude higher than in any other cell type of the human brain. We postulate that this underpins selective vulnerability and thus clinical presentation, and that loss of PKCγ cell membrane binding, cytoplasmic aggregation and Purkinje cell death represent the morphological substrate of the SCA14 H101Q mutation in human brain.

Many neurodegenerative diseases are characterized by the formation of disease-specific inclusions including Parkinson’s Disease, Huntington’s Disease and the polyglutamine SCAs [25, 35]. Inclusion bodies are generated by aggregation of misfolded proteins and often become detergent-insoluble. To formally confirm the insolubility of the PKCγ aggregates in SCA14 cerebellum, we carried out biochemical fractionation of cerebellar tissue into Triton X-100-soluble and -insoluble fractions. PKCγ was found in both soluble and insoluble fractions in control cerebellum (Fig. 2e). In contrast, PKCγ in SCA14 cerebellum was found almost exclusively in the insoluble fraction (Fig. 2e, f). Together, these findings suggest that in SCA14 Purkinje cells, PKCγ is mislocalized and aggregated in detergent-insoluble inclusions.

### Generation of SCA14 human iPSCs

To better understand the pathological mechanisms that cause SCA14, we generated human iPSC lines from fibroblasts obtained from two patients carrying the H36R mutation and from two patients with the H101Q mutation (Additional file 1: Figure S1 & S2, Suppl. Methods). At least two iPSC clones were generated from each patient. Age- and sex-matched control iPSC lines, reprogrammed using Sendai reprogramming viruses in the same laboratory, generated through the Oxford Parkinson’s Disease Centre, have been published previously [16, 18]. All iPSC lines displayed embryonic stem cell-like morphology and expressed the pluripotency-associated proteins Tra-1-60 and Nanog (Additional file 1: Figure S2B & D). Clearance of viral transgenes was confirmed by qRT-PCR (Additional file 1: Figure S2C). Genome integrity was confirmed by Illumina SNP arrays (Additional file 1: Figure S2E). *PRKCG* genotypes were confirmed in all quality-checked iPSC lines by Sanger sequencing (Additional file 1: Figure S2F).

### SCA14 mutations cause PKCγ aggregation in human iPSCs

**Fig. 3:**
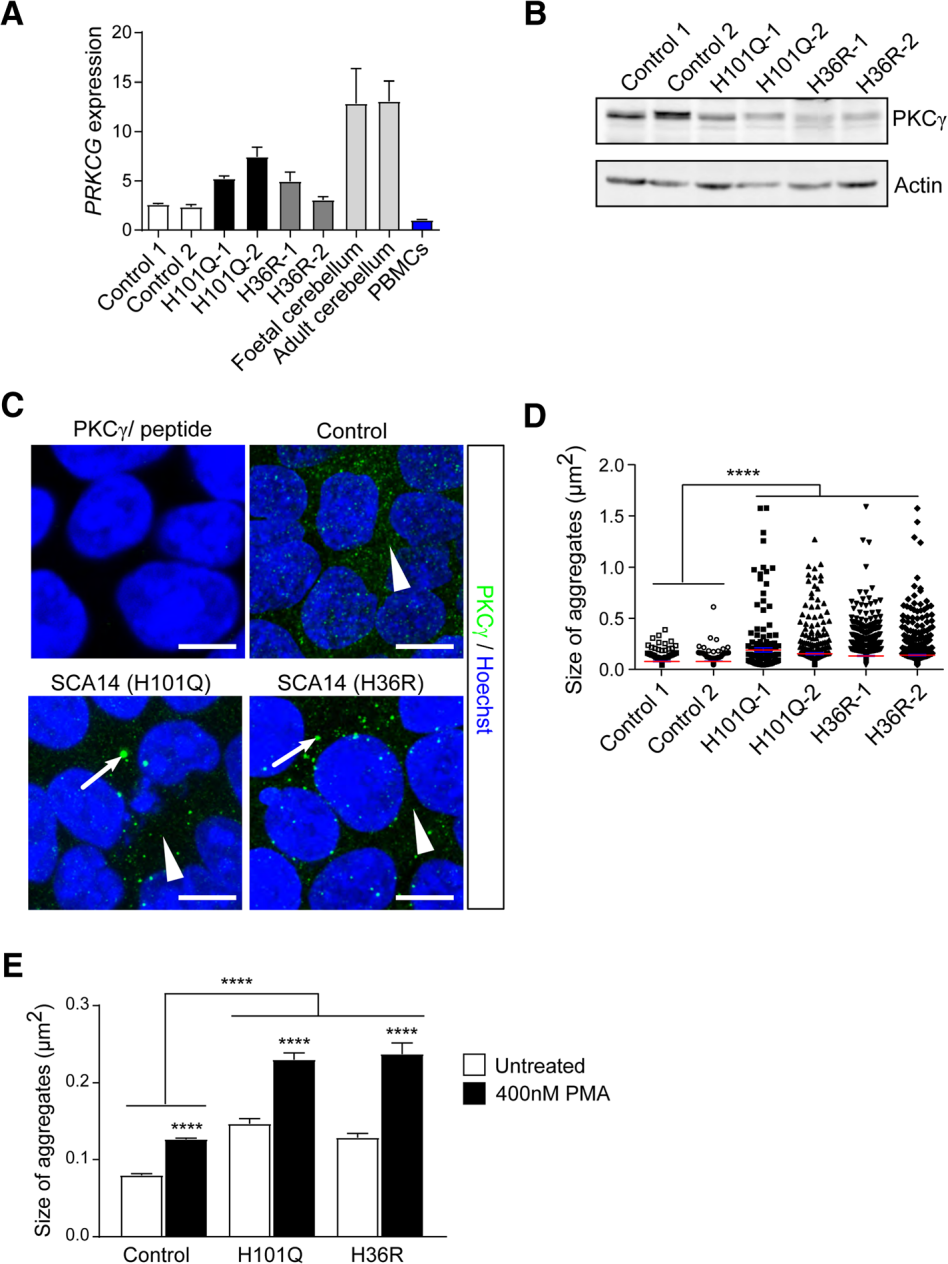
Mutant PKCγ forms cytoplasmic aggregates in iPSCs. **a** *PRKCG* mRNA expression in control and patient iPSC lines. RNA extracted from fetal and adult human cerebellum was included as positive controls. *PRKCG* is not expressed in peripheral blood mononuclear cells (PBMCs) according to data from GTEx, BioGPS, and CGAP SAGE, and thus, RNA extracted from PBMCs was used as negative control. *PRKCG* gene expression levels were normalized to housekeeping gene β-actin, and are shown relative to negative control. **b** PKCγ protein expression in control and patient iPSC lines**.** Actin: loading control. **c** Immunostaining of iPSC lines for PKCγ. Specificity of the anti-PKCγ antibody was confirmed by peptide absorption assay (top left panel). Small punctate staining of PKCγ (white solid arrowheads) was observed in the cytoplasm of control iPSCs and SCA14 iPSCs, while large cytoplasmic aggregates (white arrows) were only present in SCA14 iPSCs. Cell nuclei are visualized by Hoechst staining. Scale bar: 10 μm. **d** PKCγ formed significantly larger aggregates in SCA14 iPSCs compared to control iPSCs (*n* = 3, ****p < 0.0001, ANOVA followed by Bonferroni’s post-hoc test). **e** Treatment with 400 nM PMA, a potent PKCγ activator, both wildtype and mutant PKCγ aggregates increased in size. Compared to control, PKCγ formed significantly larger aggregates in SCA14 iPSCs following PMA treatment (*n* = 3, ****p < 0.0001, two-way ANOVA followed by Bonferroni’s post-hoc test)

Although PKCγ is generally known to be a neuron-specific kinase, we identified robust expression of *PRKCG* RNA in both control and patient iPSCs human iPSCs (Fig. 3a, b), consistent with previous reports [24]. This prompted us to investigate the cellular phenotypes of iPSCs expressing mutant PKCγ. Similar to our observations in post-mortem cerebellar tissue, wildtype PKCγ was present in small cytoplasmic puncta, which partially co-localized with the cis-Golgi marker GM130, early endosomal marker EEA1 and recycling endosomal marker RAB11 (data not shown). In contrast, mutant PKCγ formed large aggregates in the cytoplasm (Fig. 3c, d), with little co-localization with Golgi and endosomal markers (data not shown). This staining pattern was observed for both SCA14 mutations, H36R and H101Q.

Prolonged activation of PKC results in its accumulation in the detergent-insoluble fraction, where it is subjected to dephosphorylation and degradation [2, 15, 33]. To address whether activation of mutant PKCγ further enhanced its aggregation, we treated control and SCA14 iPSCs with 400 nM of phorbol 12-myristate 13-acetate (PMA), a potent PKC activator. Stimulation with PMA led to a more significant increase in the size of aggregates in SCA14 patient cells compared to controls (Fig. 3e). DMSO vehicle control did not affect PKCγ aggregation (Additional file 1: Figure S3). Together, these results indicate that the SCA14 H36R and H101Q mutations cause the aggregation of PKCγ in the cytoplasm of iPSCs, which is further enhanced following PKCγ activation.

### Reduced membrane targeting of mutant PKCγ

**Fig. 4:**
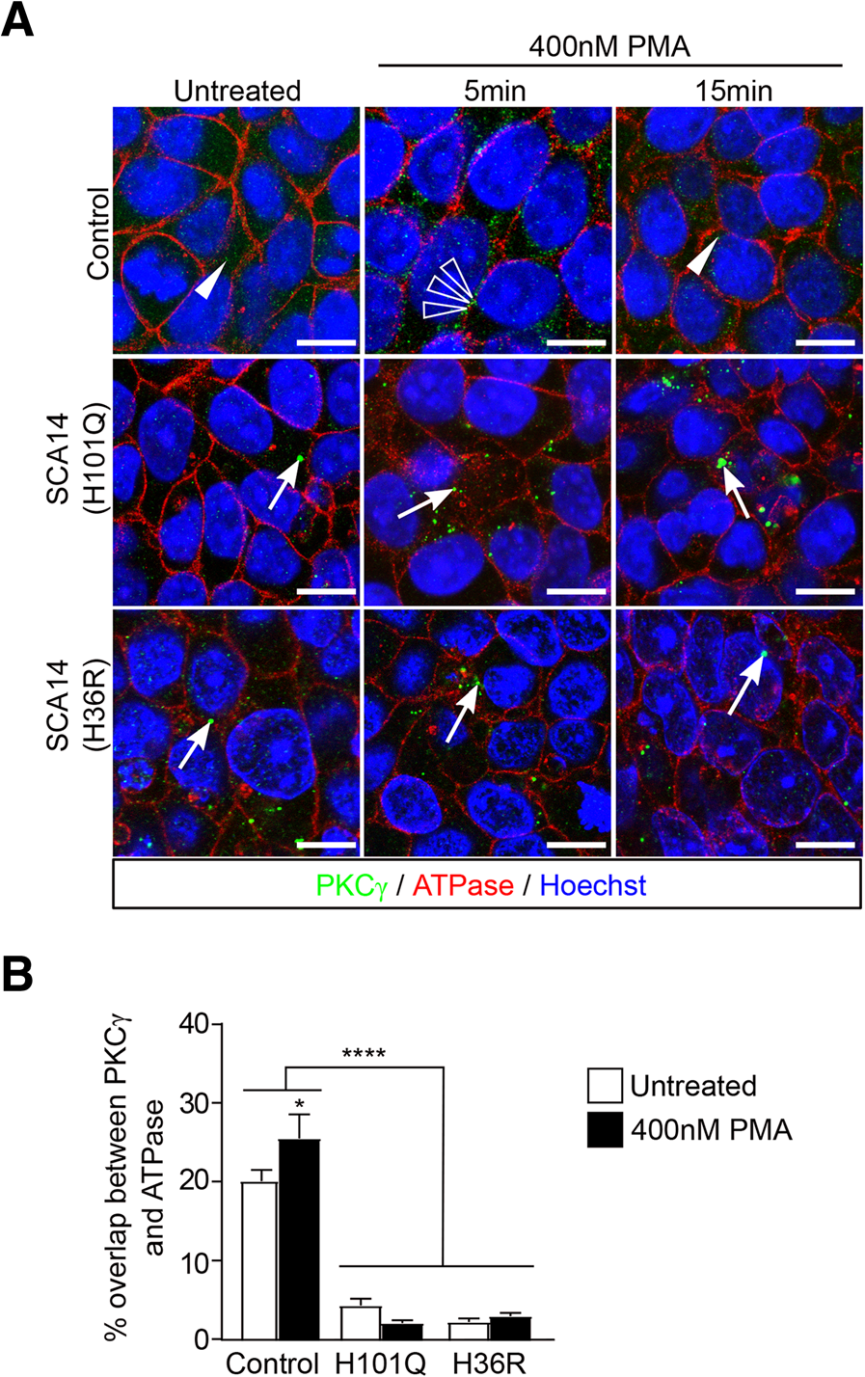
SCA14 mutations reduce PMA-induced membrane translocation of PKCγ. **a** Control and patient iPSCs were immunostained for PKCγ before or after treatment with PMA. The cell membrane was stained with an antibody against sodium potassium ATPase. Cell nuclei are visualized by Hoechst staining. In unstimulated control iPSCs, PKCγ was expressed as small dots in the cytoplasm (white solid arrowhead). After 5 min of PMA treatment, PKCγ was found at the plasma membrane (white hollow arrowheads), and returned to the cytoplasm after 15 min of PMA treatment (white solid arrowhead). In unstimulated SCA14 iPSCs, large aggregates (white arrowheads) of PKCγ were present in the cytoplasm. PKCγ inclusions remained in the cytoplasm (white arrowheads) throughout the treatment with PMA. Scale bar: 10 μm. **b** PKCγ in SCA14 iPSCs showed significantly less membrane association than in control iPSCs in response to PMA stimulation (*n* = 3, ****p < 0.0001, two-way ANOVA followed by Bonferroni’s post-hoc test)

The C1 domain mediates binding of PKCγ to DAG and phospholipids at the plasma membrane [8]. As both SCA14 mutations investigated in this study are located in the C1 domain, we next determined whether SCA14 mutants would be impaired in their membrane targeting. Interestingly, as described above, PKCγ immunostaining at the plasma membrane of Purkinje cells was markedly reduced in SCA14 post-mortem cerebellar tissue (Fig. 2d). To test whether membrane translocation of PKCγ was affected in patient iPSCs, cells were treated with 400 nM PMA to activate PKCγ. In control iPSCs, PKCγ co-localization with sodium potassium ATPase at the plasma membrane increased after 5 min of PMA treatment, and after 15 min of PMA treatment, PKCγ was found again in the cytoplasm (Fig. 4a, b). In contrast, mutant PKCγ remained aggregated in the cytoplasm and did not translocate to the plasma membrane in response to PMA treatment (Fig. 4a, b). Similar results were obtained following treatment with phorbol 12,13-dibutyrate (PDBu), an alternative PKCγ-activating phorbol ester (Additional file 1: Figure S4A). Together, these findings indicate that mutant PKCγ is impaired in its ability to translocate to, or be retained at, the plasma membrane.

### Impaired degradation of SCA14 PKCγ aggregates

We next investigated the cellular responses to mutant and aggregated PKCγ. Cells operate two major protein degradation machineries: the ubiquitin proteasome system (UPS) and autophagy [14]. Impairment of both the UPS and autophagy have been associated with neurodegenerative disorders [14, 31]. This is further supported by the accumulation of intraneuronal aggregates of misfolded proteins in many neurodegenerative disorders [25, 31, 35]. Most of these aggregates are visible with light microscopy with immunohistochemistry against the disease-defining protein species (e.g. alpha-synuclein, C-terminal huntingtin) and components of the ubiquitin proteasome or macroautophagy systems. Interestingly, we did not find co-localization of the PKCγ aggregates with antibodies to ubiquitin or p62 in SCA14 cerebellum (data not shown). We next assessed whether mutant PKCγ aggregates in iPSCs were tagged with ubiquitin in an attempt by the cells to clear the aggregates. Control and patient iPSCs were immunostained with antibodies against PKCγ or ubiquitin in the presence or absence of PMA or PDBu. No ubiquitin-positive PKCγ aggregates were identified (Additional file 1: Figure S4), consistent with the results obtained in post-mortem SCA14 cerebellum.

**Fig. 5:**
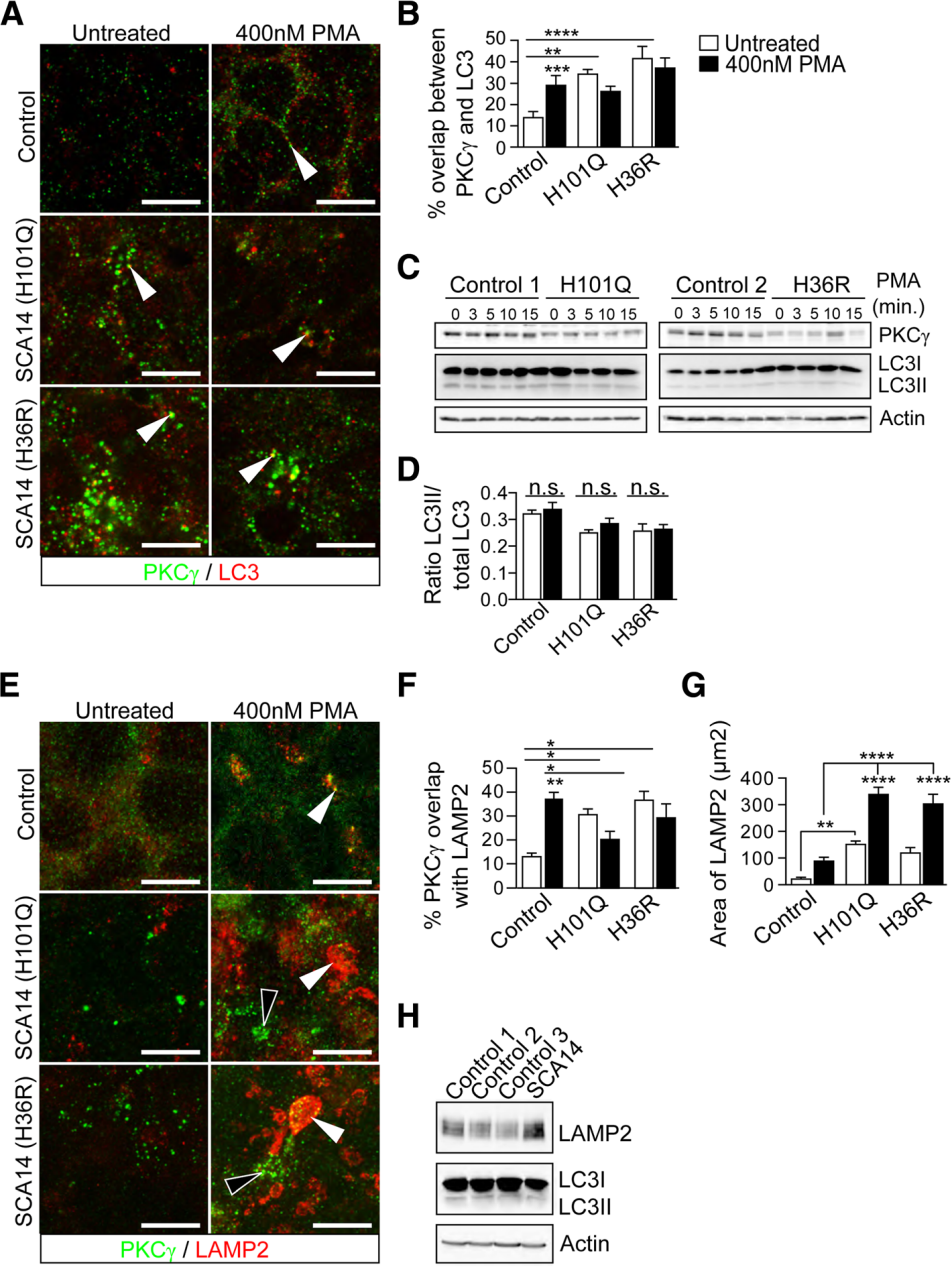
Impaired degradation of SCA14 PKCγ aggregates. **a**, **b** Control and patient iPSCs were immunostained for PKCγ and the autophagosomal marker LC3 before or after treatment with PMA for 15 min. In control iPSCs, PKCγ co-localization with LC3 (white solid arrowheads) increased upon treatment with PMA. In untreated SCA14 iPSCs, there was already a significant overlap with LC3 (white solid arrowheads), which did not further increase upon PKCγ activation (*n* = 3, **p < 0.01, ***p < 0.001, ****p < 0.0001, two-way ANOVA followed by Bonferroni’s post-hoc test). **c** Lysates of iPSCs were subjected to immunoblotting for PKCγ and LC3. LC3I represents free cytosolic cleaved LC3. LC3II represents LC3 that is anchored to the autophagosome membrane and indicates autophagosome load. **d** Ratio of LC3II/total LC3 levels remained constant in control and SCA14 iPSCs following PMA treatment. **e**, **f** Control and patient iPSCs were immunostained for PKCγ and the lysosomal marker LAMP2 before or after treatment with PMA for 15 min. In control iPSCs, co-localization of PKCγ with LAMP2 increased upon activation (white solid arrowheads). In SCA14 iPSCs, by contrast, lysosomes fused together into larger vesicles enclosing PKCγ aggregates (white arrowheads) in the presence of PMA. However, the majority of PKCγ aggregates did not co-localize with LAMP2-postive lysosomes (white hollow arrowheads) (*n* = 3, *p < 0.05, **p < 0.001, two-way ANOVA followed by Bonferroni’s post-hoc test*)*. **g** The area of LAMP2 signal, representing the formation of lysosomes, significantly increased in both control and SCA14 iPSCs following PMA treatment. The lysosomal area was significantly larger in SCA14 iPSCs compared to control iPSCs (*n* = 3, **p < 0.01, ****p < 0.0001, two-way ANOVA followed by Bonferroni’s post-hoc test). **h** Cerebellar lysates were subjected to immunoblotting for LAMP2 and LC3. LC3I represents free cytosolic cleaved LC3. LC3II represents LC3 that is anchored to the autophagosome membrane and indicates autophagosome load

The absence of PKCγ ubiquitination led us to investigate whether mutant PKCγ aggregates might be degraded through a different cellular pathway. We first looked at the formation of autophagosomes using immunostaining microtubule-associated protein 1 light chain 3 (LC3), a central protein in the autophagy pathway. In control iPSCs, we observed a significant increase in the overlap between PKCγ and LC3 following activation by PMA or PDBu (Fig. 5a, b; Additional file 1: Figure S4). In contrast, there was already a significant overlap between SCA14 PKCγ and LC3 in unstimulated iPSCs (Fig. 5a, b). This overlap did not increase upon further PKCγ activation by PMA or PDBu (Fig. 5a, b; Additional file 1: Figure S4), despite the increased formation of PKCγ aggregates observed (Fig. 3e). Overall, autophagosome levels did not significantly change in the presence of mutant PKCγ aggregates or upon PKCγ activation (Fig. 5c, d). These results indicate that aggregated mutant PKCγ is not cleared efficiently by the autophagosome in SCA14 iPSCs.

Aggregated proteins that are engulfed by autophagosomes are subsequently degraded through the fusion with lysosomes [31]. Alternatively, aggregated proteins can also be degraded by lysosomes through autophagosome-independent pathways [14]. We found that a small proportion of wildtype PKCγ co-localized with the lysosomal marker LAMP2 (lysosome-associated membrane protein 2) in the absence of PMA treatment (Fig. 5e, f). Following PKCγ activation in control iPSCs, both the lysosomal area, and the co-localization of PKCγ and LAMP2 significantly increased (Fig. 5f, g; Additional file 1: Figure S4). In SCA14 iPSCs, a significant enlargement of the lysosomal compartment and co-localization of PKCγ with LAMP2 was already observed prior to PMA treatment. (Fig. 5e-g). Upon PMA treatment, lysosomes fused together and formed very large vesicles (Fig. 5e, g; Additional file 1: Figure S4). Some PKCγ aggregates were found to be enclosed within these large lysosomes. However, the majority of mutant PKCγ did not co-localize with LAMP2 (Fig. 5e, f). Moreover, compared to control iPSCs, less PKCγ was found to co-localize with LAMP2 following activation in SCA14 iPSCs (Fig. 5f). Together, these results suggest that despite lysosomal enlargement, aggregated mutant PKCγ is not efficiently targeted by lysosomes and thus accumulates as cytosolic aggregates in SCA14 iPSCs. Interestingly, increased expression of LAMP2 but no change in LC3 levels were also found in SCA14 cerebellum (Fig. 5h) indicating that the findings in SCA14 iPSCs reflect cerebellar pathology.

### Increased PKCγ kinase activity in SCA14 patient cells

**Fig. 6:**
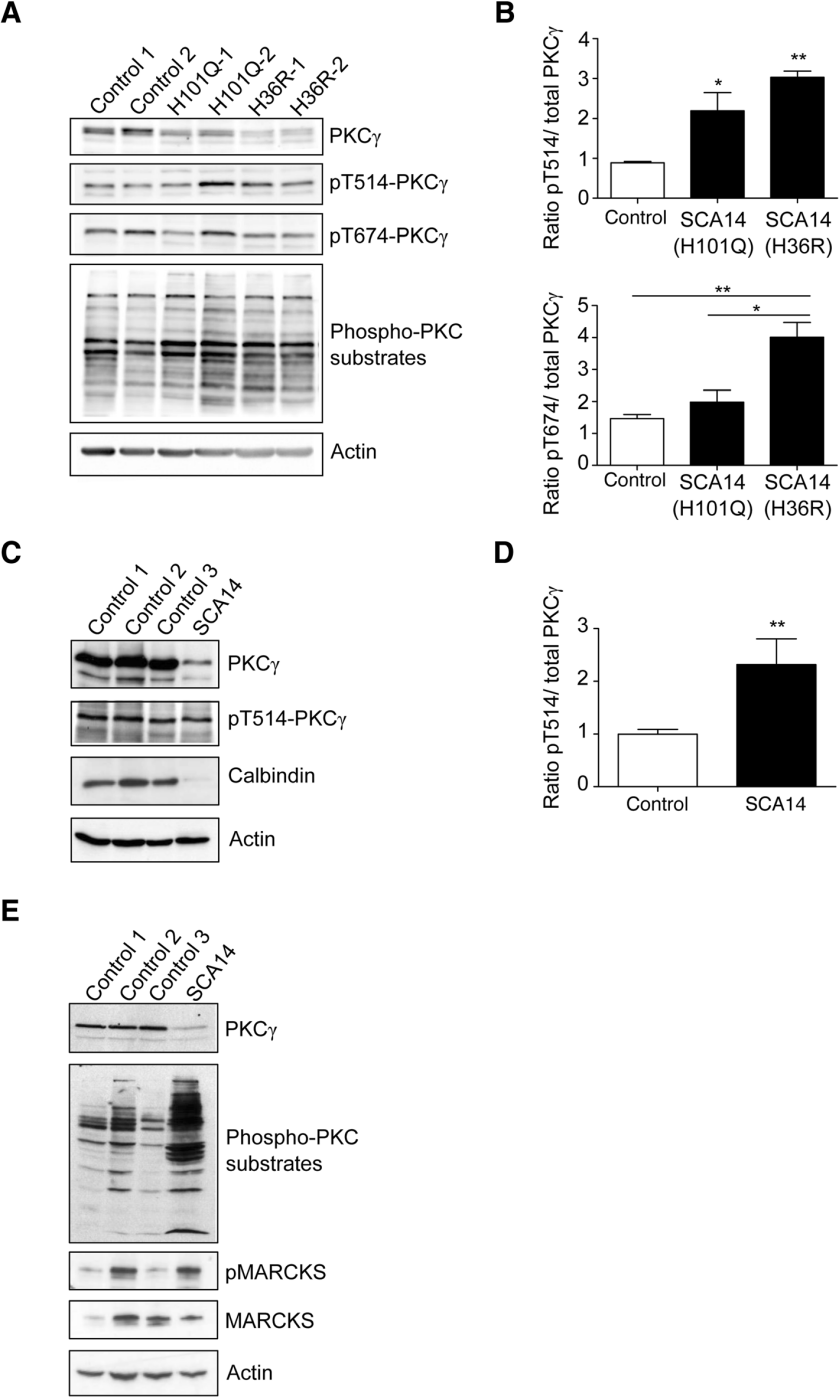
Increased PKC kinase activity in SCA14 patient cells. **a** Lysates of iPSCs were subjected to immunoblotting for PKCγ, T514- and T674-phosphorylated PKCγ and phospho-PKC substrates. Actin was used as loading control. **b** Quantification of PKCγ phosphorylation at T514 (upper panel) and T674 (lower panel) versus total PKCγ. Phosphorylation was significantly increased in SCA14 iPSCs compared to controls (*n* = 3, *p < 0.05, **p < 0.01, ANOVA followed by Bonferroni’s post-hoc test). **c** Lysates from post-mortem cerebellum were subjected to immunoblotting for PKCγ, T514-phosphorylated PKCγ, Calbindin and Actin. **d** Quantification of PKCγ phosphorylation at T514 versus total PKCγ. T514 phosphorylation was significantly increased in SCA14 (H101Q) cerebellum compared to controls (*n* = 3, **p < 0.01, unpaired students’ t-test). **e** Cerebellar lysates were subjected to immunoblotting for PKCγ, phospho-PKC substrates, phosphorylated (p) MARCKS, MARCKS and Actin

Phosphorylation is known to play an important role in regulating PKCγ, rendering PKCγ in a catalytically competent conformation, and protecting it from degradation [2]. PKCγ phosphorylation occurs sequentially at three conserved residues: phosphoinositide-dependent kinase 1 (PDK1) phosphorylates PKCγ within the activation loop (T514), and autophosphorylation occurs within the turn motif (T655) and the hydrophobic motif (T674) at the C-terminal tail (Fig. 1a). Having identified that mutant PKCγ is not efficiently cleared in SCA14 patient cells, we next determined whether its phosphorylation status might be altered compared to wildtype PKCγ, which might affect its stability and kinase activity. Although overall PKCγ expression was lower in SCA14 iPSCs than control cells (Figs. 4b & 6a), PKCγ was highly phosphorylated at T514 and T674 in SCA14 iPSCs as determined by immunoblotting with phospho-specific antibodies (Fig. 6a, b). We also analyzed the phosphorylation status of PKCγ in SCA14 (H101Q) cerebellar tissue. Less PKCγ protein was present in SCA14 cerebellum compared to controls (Fig. 6c). We noted a similar reduction in Calbindin protein levels, consistent with the loss of Purkinje cells in the SCA14 cerebellum that was observed histopathologically (Fig. 2b). Despite the reduction in total PKCγ protein level, there was no reduction in phosphorylation levels of the PKCγ activation loop (Fig. 6c). Quantification of the phosphoT514- PKCγ levels in three independent experiments showed that net phosphorylation of PKCγ was significantly increased in SCA14 cerebellum compared to control tissue (Fig. 6d). Together with previous results, these findings indicate that the SCA14 mutations H36R and H101Q promote the aberrant maturation of PKCγ into a catalytically competent and stable conformation.

Phosphorylated PKCγ increases its affinity for Ca^2+^ and promotes substrate binding [2, 8]. We therefore next asked whether the SCA14 mutations affect downstream PKCγ signaling and assessed the phosphorylation status of several PKCγ substrates. First, we employed a pan-phospho-PKC substrate antibody that recognizes cellular proteins (Ser-)phosphorylated at PKC consensus motifs. PKC substrate phosphorylation was consistently higher in iPSCs derived from SCA14 patients than in controls (Fig. 6a). Moreover, we detected a robust increase in PKC substrate phosphorylation in the SCA14 cerebellum compared to controls (Fig. 6e). We also assessed the phosphorylation status of a well-known PKC target in the brain, myristoylated alanine-rich C-kinase substrate (MARCKS). Using a phospho-specific antibody, we detected elevated phospho-MARCKS levels in the SCA14 cerebellum compared to controls (Fig. 6e). Together, these findings suggest that the SCA14 mutations H36R and H101Q cause increased kinase activity of PKCγ in both patient iPSCs and cerebellum.

## Discussion

**Fig. 7:**
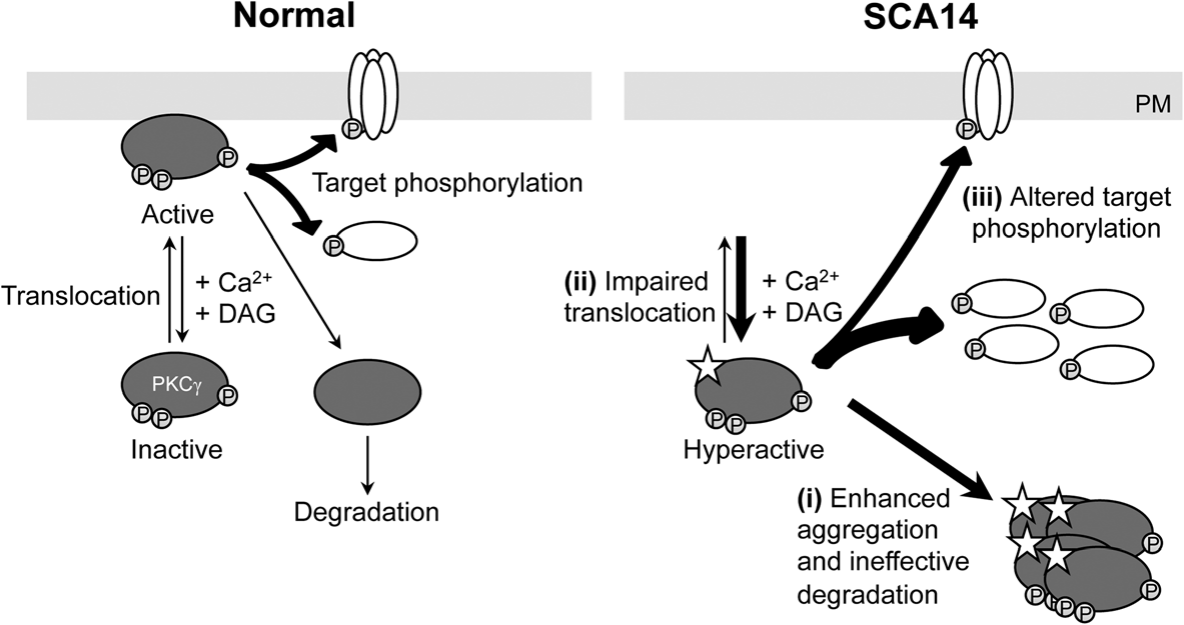
Model of the functional effect of PKCγ mutations. Normally (left panel), mature wildtype PKCγ resides in the cytosol in an autoinhibited conformation. Binding of diacylglycerol (DAG) and calcium ions (Ca^2+^) activates and promotes the translocation of PKCγ to the plasma membrane (PM), where active PKCγ phosphorylates its membrane substrates. PKCγ returns to an autoinhibited conformation (inactive) following the decay of its second messengers. The membrane-bound conformation of PKCγ is sensitive to dephosphorylation. Prolonged activation of PKCγ leads to its dephosphorylation by phosphatases. The dephosphorylated PKCγ can be tagged by ubiquitin and subsequently degraded. In contrast, in SCA14, PKCγ with mutated C1 domain adapts an open conformation and is hyper-active in the cytoplasm. (i) Highly phosphorylated mutant PKCγ forms aggregates, which accumulate in the cytoplasm due to inefficient degradation. (ii) Mutant PKCγ fails to translocation to the plasma membrane and remains in the cytoplasm. (iii) This might lead to altered phosphorylation of its substrates at the membrane and in the cytoplasm

In this study, we provide novel insights into the pathogenesis of SCA14. We present a unique in vitro model using human patient-derived iPSCs carrying two distinct SCA14 mutations in the C1 domain of PKCγ, H36R and H101Q, respectively, that recapitulate key pathological findings observed in SCA14 cerebellum. Our findings indicate that SCA14 is likely to be caused by three interconnected pathogenic mechanisms (Fig. 7): (i) SCA14 mutations in the C1 domain enhance the aggregation of PKCγ, aided by insufficient protein degradation, (ii) a reduction of mutant PKCγ at the plasma membrane is likely to decrease its interaction with target substrates, and (iii) extended cytoplasmic retention of hyper-active PKCγ results in aberrant phosphorylation of substrates in the cytoplasm.

This study sheds light on the important question of how PKCγ harboring mutations in the C1 domain causes SCA14 pathology. Mutations in other domains of PKCγ might cause disease through other or additional mechanisms. Indeed, other PKCγ mutations in overexpression studies have been reported to drive a plethora of cellular phenotypes, which often contradict each other [1, 43]. This is the first study that investigates the consequences of more physiological levels of expression of two distinct SCA14 mutations in relevant human models, iPSCs and post-mortem cerebellar tissue. Only one postmortem brain of a SCA14 patient with a H101Y mutation has been reported previously [11]. Other than the loss of Purkinje cells, little pathology was observed, likely due to the insufficient quality of the post-mortem material. Remaining Purkinje cells were shown to display markedly reduced immunoreactivity for PKCγ without any visible protein aggregation (16). In contrast, our results suggest that aggregation of PKCγ is central to SCA14 pathology. Both mutations investigated in this study cause aggregation of PKCγ in the cytoplasm in both iPSCs and in Purkinje cells of SCA14 (H101Q) cerebellum, but not in other brain regions (which show only minimal PKCγ expression in adult human brain). The aggregation of misfolded proteins is a central feature in many neurodegenerative disorders. Owing to their post-mitotic nature, neurons are particularly vulnerable to misfolded proteins as they cannot dilute toxic substances by division [14]. Moreover, in many neurodegenerative disorders components of the protein degradation machinery are impaired, a phenomenon that is further worsened as neurons age. Interestingly, we found that mutant aggregated PKCγ did not co-localize with ubiquitin. Dephosphorylation of PKC is a prerequisite of the subsequent ubiquitination and degradation via a proteasome pathway [28]. Given the hyper-phosphorylated state of mutant PKCγ, our results raise the possibility that mutant PKCγ might be resistant to ubiquitination and subsequent proteasomal or autophagic degradation, which is in contrast to a previous study employing transient overexpression of mutant PKCγ [46]. This discrepancy might be explained by the massive overexpression of mutant PKCγ that could trigger a cellular response that is different from that under physiological circumstances. We found that there was a significant overlap between PKCγ and LC3 in unstimulated SCA14 iPSCs, consistent with the idea that mutant PKCγ is already in an active and aggregated conformation [21, 42]. However, this overlap did not further increase following phorbol ester activation, despite the significant increase in aggregation size. This is consistent with the observation that autophagosomes mostly degrade non-aggregated or small aggregated proteins, but not large inclusions [31]. The enhanced formation of lysosomes in SCA14 iPSCs and cerebellum suggests that mutant PKCγ might enter the lysosomal pathway via alternative routes. Dephosphorylation- and ubiquitination-independent downregulation through lipid raft-mediated endocytic and lysosomal pathways has been described for PKCα [27, 29]. Although endosomal sequestration of mutant PKCγ has been observed in vitro [17], this could not be confirmed in our study suggesting the existence of alternative mechanisms such as chaperone-mediated autophagy via LAMP2A [14].

In our study, we found reduced staining of mutant PKCγ at the Purkinje cell membrane in SCA14 cerebellum. This is consistent with previous studies that have suggested altered translocation of mutant PKCγ to the plasma membrane following activation in heterologous cell lines [1, 42] and primary Purkinje cells [39]. PKC has long been implicated in the regulation of neurotransmission and synaptic plasticity by phosphorylating membrane receptors and ion channels [8]. Mutant PKCγ might affect membrane excitability in Purkinje cells either indirectly through altering the membrane kinetics of PKCα [39] or directly via phosphorylation of critical receptors. Specific physiological targets of PKCγ remain largely unknown, and their identification might provide important clues about the selective vulnerability of Purkinje cells in SCA14. One possible membrane-associated substrate of PKCγ might be the C3-type transient receptor potential (TRPC3) channel, which is highly expressed in Purkinje cells [19]. In Purkinje cells, TRPC3 is activated downstream of mGluR1 signaling, resulting in calcium influx [19]. Interestingly, PKCγ, which is also activated downstream of mGluR1 [23], has been shown to negatively regulate calcium entry via phosphorylation of TRPC3 [40, 41]. Moreover, transiently overexpressed PKCγ mutants failed to phosphorylate TRPC3 despite their high catalytic activity [1]. Thus, a failure of mutated PKCγ to phosphorylate and inhibit TRPC3 might lead to excessive calcium influx upon TRPC3 activation and might thereby contribute to Purkinje cell dysfunction and cell death. Abnormal TRPC3 signaling is likely to be a common pathological mechanisms in different subtypes of ataxia [4, 30], suggesting a pathological role for PKCγ beyond SCA14. Indeed, *Trpc3* and *Prkcg* were recently identified as hub genes in gene networks misregulated in mouse models of SCA1 [20] and SCA2 [34].

Together, these findings suggest that SCA14 pathogenesis might be partially explained by a loss-of-function of PKCγ at the cell membrane. However, the absence of SCA14-related phenotypes in *Prkcg* knockout mice suggests that the disease cannot be fully explained by a reduction of PKCγ function. Interestingly, we found an increase in PKC substrate phosphorylation in SCA14 cerebellum and iPSCs. Increased kinase activity of mutant PKCγ has also been reported in in vitro studies [1, 3, 43], supporting and support the hypothesis that mutations in the C1 domain facilitate the ligand-induced ‘open’ and signaling competent conformation of PKCγ. Recently, increased PKC kinase activity has been shown to be neuroprotective in mouse models of SCA1 and SCA2 [13]. The identities of the phosphorylated PKC targets in these conditions compared to SCA14 remain to be elucidated. Similarly, it will be important to test whether the same aberrantly phosphorylated targets are found in both SCA14 cerebellum and iPSCs. Moreover, it is conceivable that PKC isoforms other than PKCγ contribute to the observed increased PKC substrate phosphorylation. Human stem cells express both canonical and non-canonical PKC isoforms [24] and Purkinje cells have been shown to express PKCα, PKCγ, PKCδ and PKCε [45].

## Conclusions

Our study is the first to describe the functional neuropathology of SCA14 in post-mortem cerebellum as well as in human iPSCs derived from patients with SCA14 mutations. Unexpectedly, PKCγ aggregation, mislocalization and increased kinase activity that we observed in SCA14 cerebellum were reproduced in SCA14 iPSCs. Purkinje cells are particularly vulnerable in SCA14, likely due to their high expression of PKCγ and its specific targets that regulate the calcium homeostasis and the unique physiological properties of these neurons. While the latter cannot be modelled in undifferentiated stem cells, the fact that patient iPSCs express PKCγ and recapitulate key pathological findings observed in SCA14 cerebellum underscores their potential as relevant tools for disease modeling and drug discovery, in addition to future studies in which SCA14 iPSCs will be differentiated to Purkinje cells.

## Additional file

Additional file 1: Supplementary Material. (PDF 3471 kb)

## Abbreviations

DAG: Diacylglycerol
iPSCs: Induced pluripotent stem cells
LAMP2: Lysosome-associated membrane protein 2
LC3: Microtubule-associated protein 1 light chain 3
MARCKS: Myristoylated alanine-rich C-kinase substrate
PDBu: Phorbol 12, 13-dibutyrate
PKCγ: Protein kinase C gamma
PMA: Phorbol 12-myristate 13-acetate
SCA14: Spinocerebellar ataxia type 14
UPS: Ubiquitin proteasome system
