# Supplementary material for: Neurodegeneration in SCA14 is associated with increased PKCγ kinase activity, mislocalization and aggregation
Source: Acta Neuropathol Commun. 2018 Sep 24;6:99. doi: 10.1186/s40478-018-0600-7 (PMC6151931; doi:10.1186/s40478-018-0600-7)
Supplement: Supplementary file 1 — Supplementary Material. (PDF 3471 kb) [file 40478_2018_600_MOESM1_ESM.pdf]

Supplementary Methods

Reprogramming of donor fibroblasts to iPSCs using Sendai virus

Reagents were from ThermoFisher (Invitrogen), unless stated otherwise. Patient information is listed in Supplementary Figure 2A. Reprogramming of iPSCs from patients SCA14-1 and SCA14-2 using SeVdp(KOSM)302L Sendai virus system (Nishimura *et al.*, 2011; Nishimura *et al.*, 2013) was carried out as previously described (Watson *et al.*, 2018). Reprogramming of skin fibroblasts from patients PRKCG01 and PRKCG02 was performed using the CytoTune®-iPS 2.0 Sendai Reprogramming Kit according to manufacturer's protocols. Briefly, patients' skin fibroblasts were thawed and expanded. Two days prior to viral transduction, the fibroblasts were detached and seeded at a range of densities on 12-well plates ($0.75x10^5$, $1x10^5$, $1.25x10^5$, $1.5x10^5$, $2.0x10^5$) and grown for two days to 90% confluency. Sendai virus was added to fibroblasts on day 0 at a multiplicity of infection (MOI) of 3 per $1.0x10^5$ cells and incubated overnight. Virus was removed the next day. A well of untransfected fibroblasts was kept for each density for monitoring cell division. Transduced fibroblasts were cultured in fibroblast medium [ADMEM, Gibco; 10% foetal calf serum; 1% Glutamax; Gibco; 1% penicillin/streptomycin (P/S)]. 8000 reprogrammed cells per ml were transferred to a layer of mouse embryonic feeder cells (CF1 MEF) (Merck) on 0.1% gelatin-coated 6-well plates and cultured in KOSR medium [KnockOut Dulbecco's modified Eagle's medium (DMEM), 10% KnockOut Serum Replacement; 0.1% Non-Essential Amino Acids, 2 mM GlutaMax-I, 0.5 mM β-mercaptoethanol, 10 ng/ml Basic Fibroblast Growth Factor (R&D), 1% P/S] on day 7. The remaining cells were pelleted as positive control for the Sendai virus clearance assay. Half of the media were changed every two days until day 13. MEF cells were set up in T-75 flasks on day 7 and cultured in KOSR medium. The medium was collected every day for seven days as conditioned medium to feed iPSCs on feeder cells after sterilization. Reprogrammed cells were fed with conditioned medium supplemented with 10 ng/ml bFGF for around 2 weeks. Colonies that emerged at around day 26 were ready for manual passaging onto Matrigel (Corning)-coated 12-well plates for culture under feeder-free condition. For passage number 5 to 8, two vials of iPSCs ($2\text{-}3x10^6$ cells each) were frozen and the remaining well were expanded. Three clones of iPSCs were expanded in mTeSR medium (StemCell Technologies) supplemented with 1% P/S for three to four weeks until reaching six 10-cm dishes. Cells were detached using 0.5 mM EDTA or pre-warmed TrypLE. iPSCs were then frozen in freezing medium containing 10% DMSO and 30% foetal bovine serum ($2\text{-}3x10^6$ cells per vial), and then stored in liquid nitrogen for long term. Three cell pellets per clone were prepared and stored at -80°C at the time of freezing for genomic DNA and total RNA preparation. iPSC expansion and cell pelleting was repeated if clones showed abnormal karyotypes. DNA was prepared using DNeasy Blood & Tissue Kit (Qiagen). Genome integrity analysis was performed using Illumina Human OmniExpress-24.v1.1 DNA Analysis Kit (WG-315-1101). The results were analysed using KaryoStudio 1.3 and GenomeStudio software (Illumina). iPSC line SNP profiles were compared to the original pool of fibroblasts, which confirmed the identity of the iPSC to the original fibroblasts. iPSC lines were also assessed for pluripotency markers by flow cytometry as previously described (Watson *et al.*, 2018). iPSC lines tested negative for mycoplasma using MycoAlert (Lonza). RT-qPCR was performed to confirm the clearance of Sendai virus and transgenes. Genotypes of human dermal fibroblasts (HDF) and iPSCs were confirmed by Sanger sequencing. Regions of exon 1 and exon 4 of the *PRKCG* gene were amplified using the Expand High Fidelity PCR System (Sigma). PCR products were purified using the QIAquick PCR Purification Kit (Qiagen) and sent for Sanger sequencing. Primers are listed in Supplementary Table 2.

Supplementary Figure 1

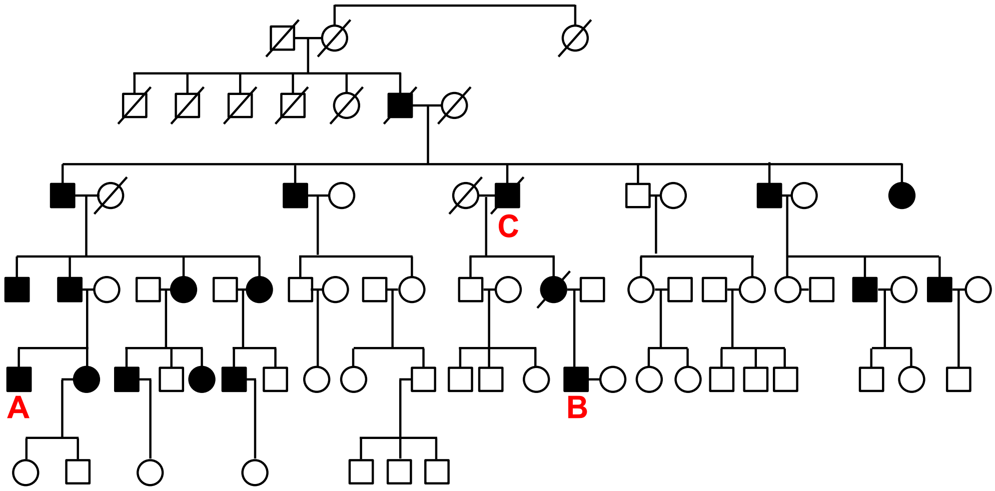

SCA14 H101Q Family Pedigree

Three patients A, B and C (in red) from a four-generation British family with the H101Q mutation were included in this study. Skin fibroblasts were obtained from patients A and B. Brain MRI was performed on patient B. Post-mortem cerebellar tissue was obtained from patient C, who died of 'natural causes' at the age of 90. Unaffected individuals are labelled in white while affected individuals are in black. Diagonal lines indicate deceased individuals. Circle: female; square: male.

Supplementary Figure 2

A

Mutation	Donors	Age	Sex	Reprogramming method	iPSC clones generated
H36R (exon 1)	PRKCG01	71	F	Cytotune 2.0	PRKCG01-1<br>PRKCG01-18
	PRKCG02	44	M	Cytotune 2.0	PRKCG02-3<br>PRKCG02-12
H101Q (exon 4)	SCA14-1	47	M	SeVdpmir302L virus	SCA14-1-1<br>SCA14-1-10
	SCA14-2	71	M	SeVdpmir302L virus	SCA14-2-3<br>SCA14-2-14
Control	AH017	67	F	SeVdpmir302L virus	AH017-3
Control	OX3	49	M	SeVdpmir302L virus	OX3-7

B

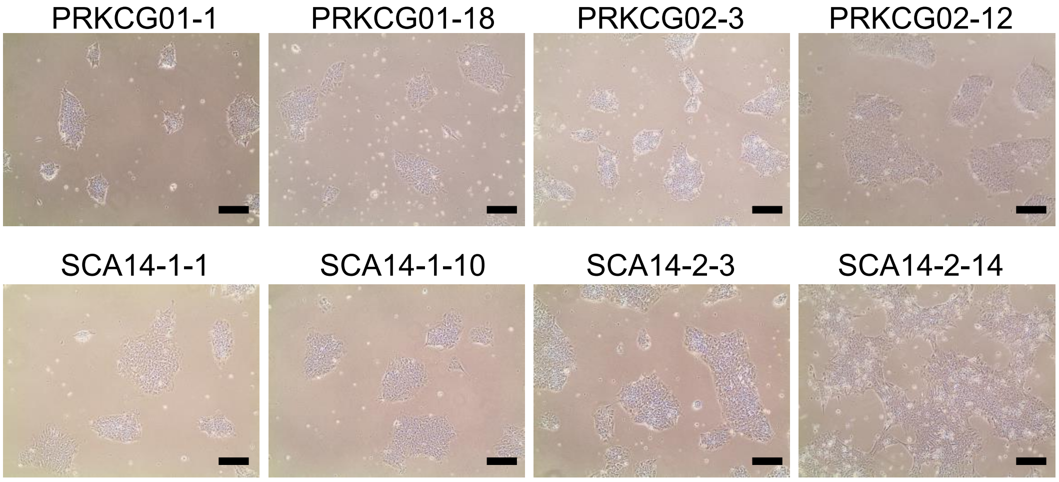

C

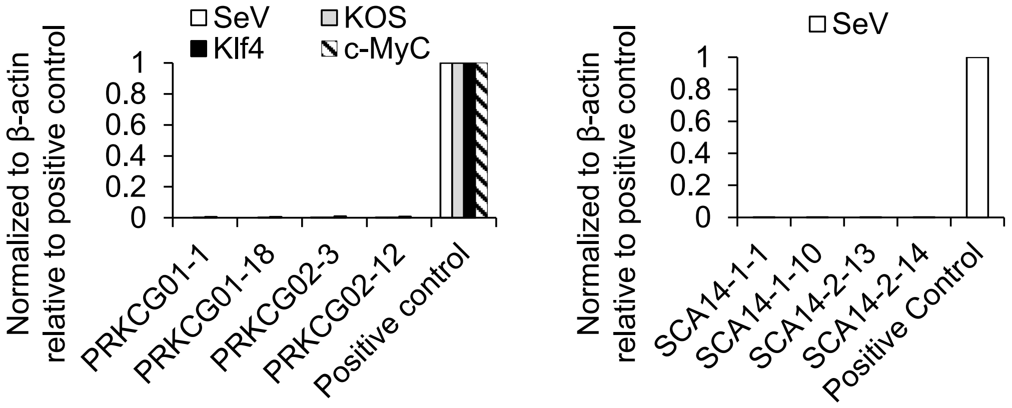

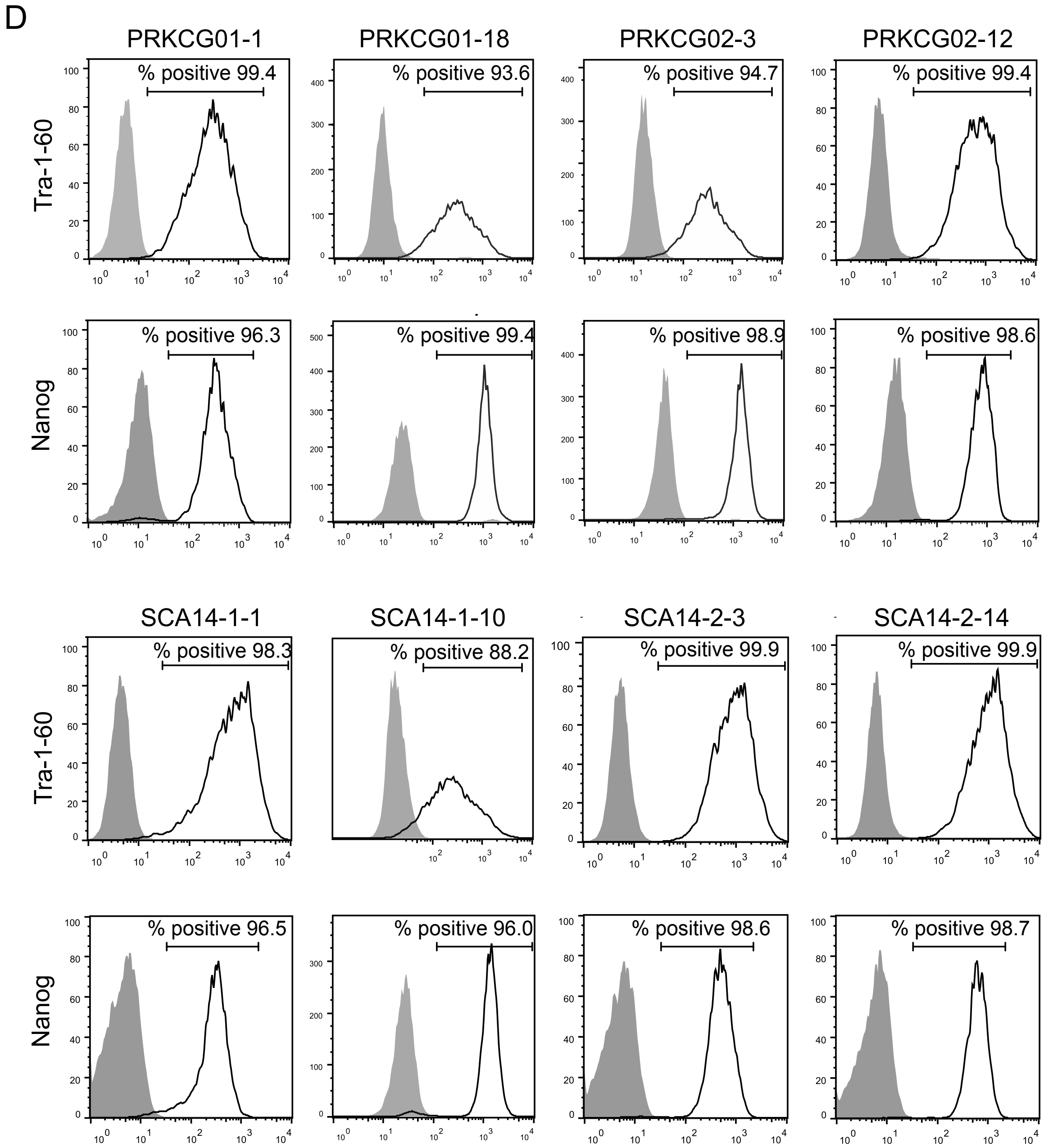
D
PRKCG01-1
PRKCG01-18
PRKCG02-3
PRKCG02-12
Tra-1-60
% positive 99.4
% positive 93.6
% positive 94.7
% positive 99.4
Nanog
% positive 96.3
% positive 99.4
% positive 98.9
% positive 98.6
SCA14-1-1
SCA14-1-10
SCA14-2-3
SCA14-2-14
Tra-1-60
% positive 98.3
% positive 88.2
% positive 99.9
% positive 99.9
Nanog
% positive 96.5
% positive 96.0
% positive 98.6
% positive 98.7

E

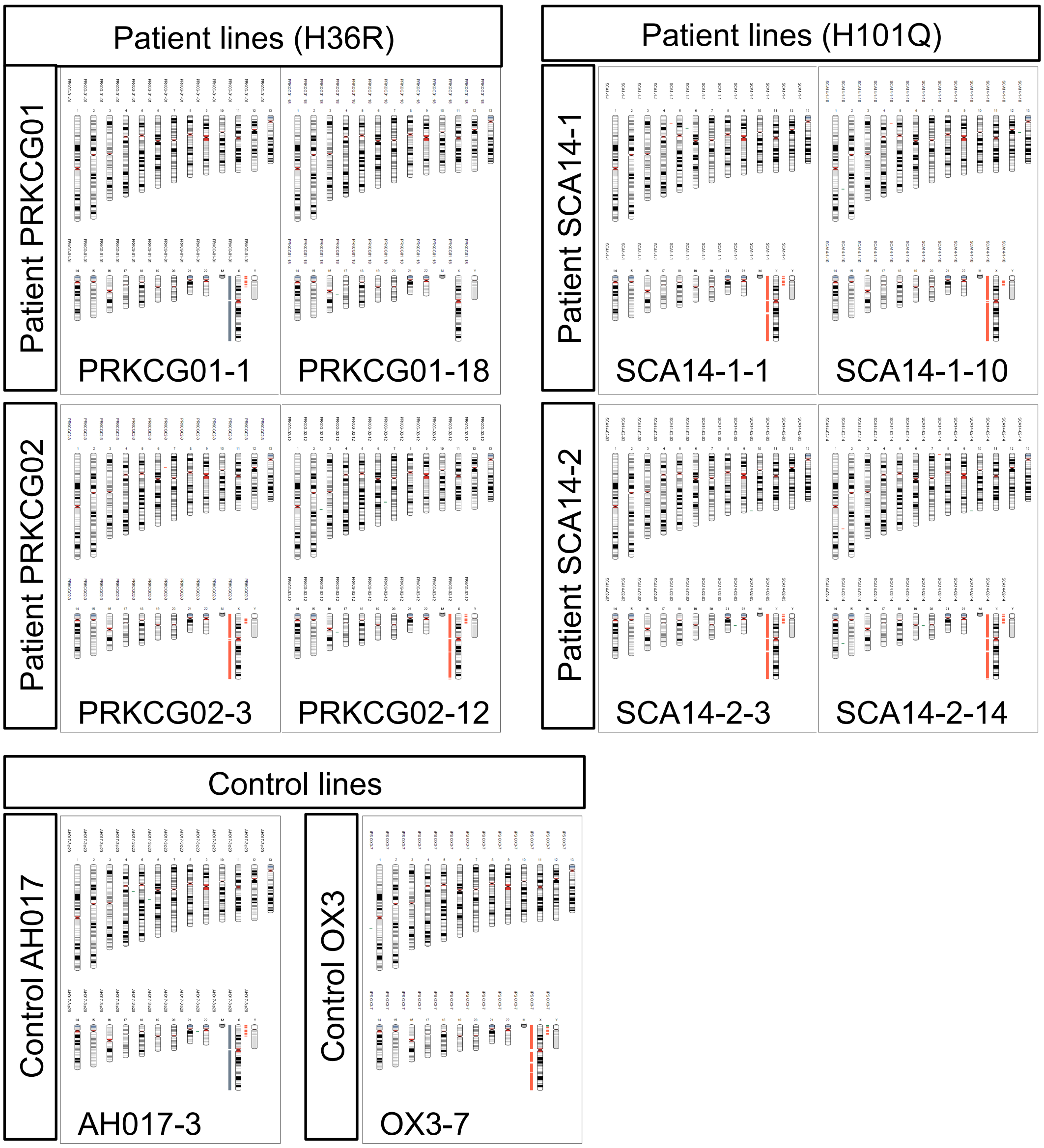
Patient lines (H36R)
Patient lines (H101Q)
Patient PRKCG01
PRKCG01-1
PRKCG01-18
Patient SCA14-1
SCA14-1-1
SCA14-1-10
Patient PRKCG02
PRKCG02-3
PRKCG02-12
Patient SCA14-2
SCA14-2-3
SCA14-2-14
Control lines
Control AH017
AH017-3
Control OX3
OX3-7

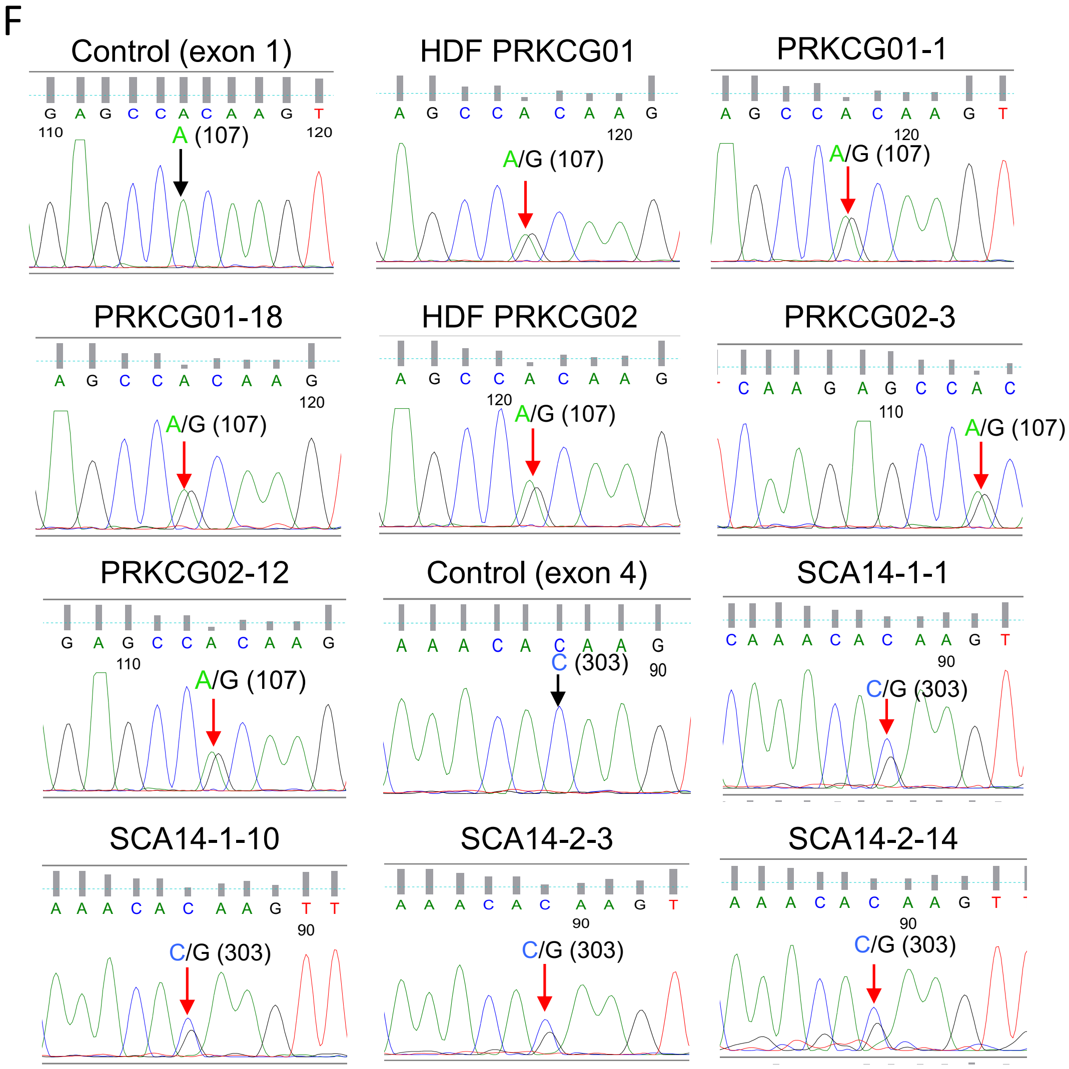

Reprogramming and quality control of SCA14 iPSCs

(A) A list of patient iPSC lines generated and control iPSC lines included in this study. Age reflects age at which skin biopsy was taken. (B) Representative phase-contrast images of patient iPSC clones cultured on Matrigel-coated surface, which showed normal iPSC morphology. (C) The expression levels of Sendai virus backbone (SeV) and transcription factors: KOS (Klf4, Oct3/4, Sox2), Klf4 and cMyc were checked using qPCR. The patient clones reprogrammed using Cytotune 2.0 were free of both viral backbone and transgenes after 13-14 passages (left). Patient clones reprogrammed using the SeVdp(KOSM)302L Sendai virus system were cleared of viral backbone after 25 passages (right). The CT values were normalised to β-actin and are shown relative to positive control (fibroblasts from one day after viral transduction). (D) Flow cytometry results for expression of the iPSC markers Tra-1-60 and Nanog. Frequency of the events in each fluorescence channel (y-axis) versus fluorescence intensity (x-axis) are displayed. Grey peaks show the data of isotype controls, while white peaks show the counts of either Tra-1-60 (FLH-1) or Nanog (FLH-4). More than 85% of the cells of each patient iPSC clone were positive for both pluripotency markers. (E) The karyograms of eight patient iPSC clones as well as two control iPSC lines are shown. All showed normal karyotypes. Amplifications (green), deletions (orange) and loss of heterozygosity regions (grey) are shown alongside the relevant chromosome.. Any changes larger than 1,000,000 nucleotides were considered abnormal. In females the X chromosomes are annotated in grey. Single-copy sex chromosomes are labelled in orange. (F) The genotypes of the patient iPSC lines were confirmed by Sanger sequencing. Positive control

showed the wildtype nucleotides at nucleotide 107 in exon 1 and at nucleotide 303 in exon 4 (black arrows). Patients' fibroblasts and iPSC lines of PRKCG01 and PRKCG02 contained the heterozygous an A-to-G transition at nucleotide 107 in exon 1 (red arrows). SCA14-1 and SCA14-2 patient iPSC lines contained a C-to-G transition at nucleotide 303 in exon 4 (red arrows). HDF: human dermal fibroblasts.

Supplementary Figure 3

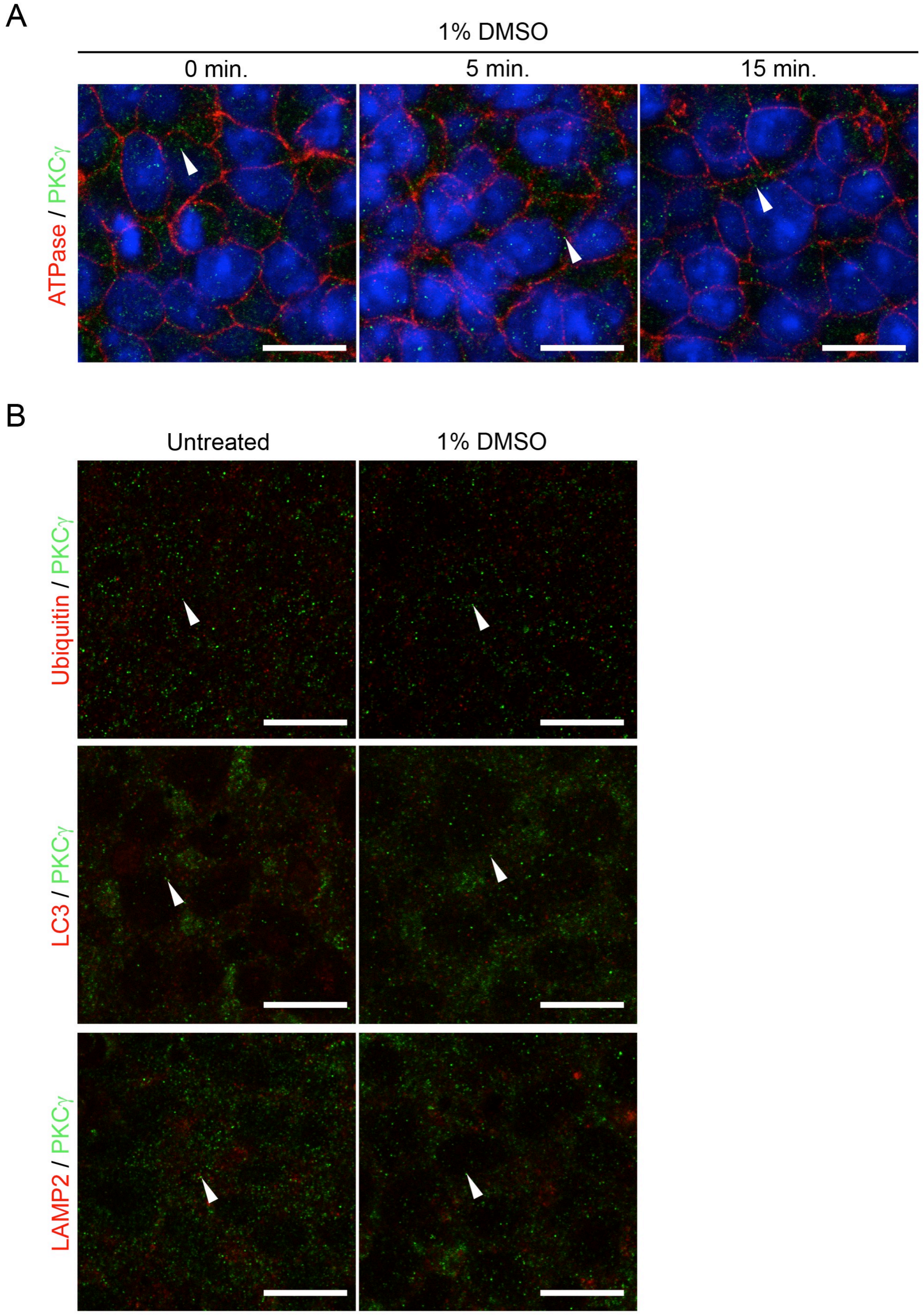

DMSO treatment does not affect iPSC cellular phenotypes

Representative images of control iPSCs co-stained with PKCγ (white solid arrowheads) and plasma membrane marker sodium potassium ATPase (**A**) and ubiquitin, autophagosome marker LC3 or lysosome marker LAMP2 (**B**), in the absence or presence of 1% DMSO. Hoechst stain was used for nuclear staining. Scale bar: 10μm.

Supplementary Figure 4

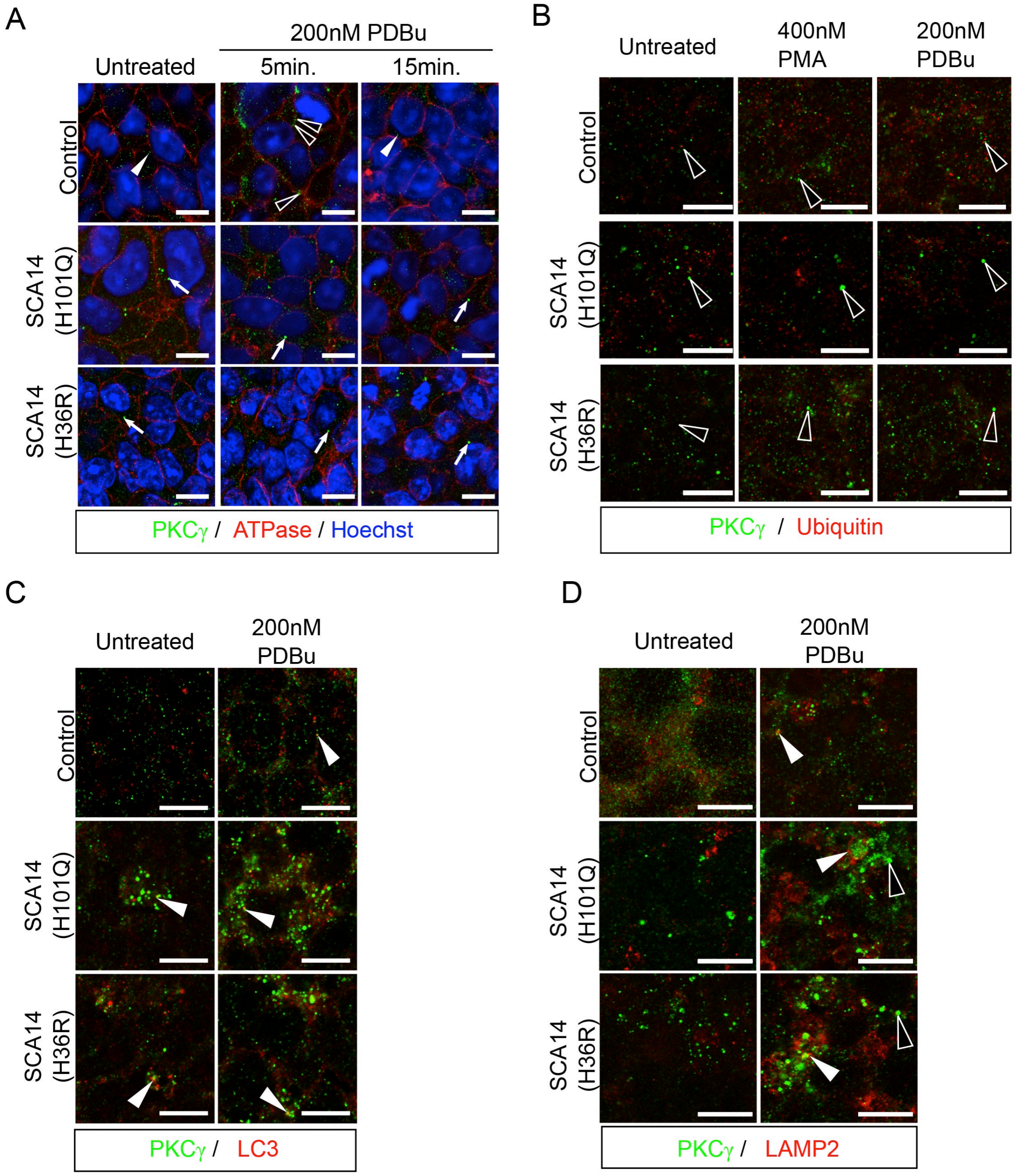

PDBu-treated iPSCs showed similar cellular phenotypes to PMA-treated cells.
(**A**) Control and patient iPSCs were immunostained for PKCγ before or after treatment with 200nM PDBu. The cell membrane was stained with an antibody against sodium potassium ATPase. Cell nuclei are visualized by Hoechst staining. In unstimulated control iPSCs, PKCγ was expressed as small dots in the cytoplasm (white solid arrowhead). After 5 min of PDBu treatment, PKCγ was found at the plasma membrane (white hollow arrowheads), and returned to the cytoplasm after 15 min of PDBu treatment (white solid arrowhead). In unstimulated SCA14 iPSCs, large aggregates (white arrows) of PKCγ were present in the cytoplasm. PKCγ inclusions remained in the cytoplasm (white arrows) throughout the treatment with PDBu. (**B**) Wildtype and mutant PKCγ was negative for ubiquitin staining (white hollow arrowheads) in

control and SCA14 iPSCs before and after PMA or PDBu treatment. (**C**) Control and patient iPSCs were immunostained for PKCγ and the autophagosomal marker LC3 before or after treatment with PDBu for 15 min. In control iPSCs, PKCγ co-localisation with LC3 (white solid arrowheads) increased upon treatment with PDBu. In untreated SCA14 iPSCs, there was already a significant overlap with LC3 (white solid arrowheads), which did not further increase with PKCγ activation. (**D**) Control and patient iPSCs were immunostained for PKCγ and the lysosomal marker LAMP2 before or after treatment with PDBu for 15 min. Co-localisation with LAMP2 is indicated by a solid arrowhead. In control iPSCs, co-localisation of PKCγ with LAMP2 increased upon activation. In SCA14 iPSCs, by contrast, lysosomes fused together into larger vesicles enclosing PKCγ aggregates (white arrows) in the presence of PDBu. However, the majority of PKCγ aggregates did not co-localise with LAMP2-postive lysosomes (white hollow arrowheads). Hoechst stain was used for nuclear staining. Scale bar: 10µm.

Supplementary Table 1. List of primers used in this study.

Gene	Forward Primer (5'-3')	Reverse Primer (5'-3')	Applications
β-actin	GCCGCCAGCTCACCATGGATG	CCATCACGCCCTGGTGCCTGG	qPCR
SeV	GGATCACTAGGTGATATCGAGC	ACCAGACAAGAGTTTAAGAGATATGTATC	qPCR
KOS	ATGCACCGCTACGACGTGAGCGC	ACCTTGACAATCCTGATGTGG	qPCR
Klf4	TTCCTGCATGCCAGAGGAGCCC	AATGTATCGAAGGTGCTCAA	qPCR
c-Myc	TAACTGACTAGCAGGCTTGTCG	TCCACATACAGTCCTGGATGATGATG	qPCR
SeV	AGACCCTAAGAGGACGAAGACAGA	ACTCCCATGGCGTAACTCCATAG	qPCR
PRKCG exon 1	AGAAAGGCAGGATCCTGGTC	CGGCGTGATAGGAGTCTGCA	PCR
PRKCG exon 4	GCTGACCTAGAGAGCAAGGC	CTTTGGAAGGGCCCTGGCA	PCR
PRKCG	GCTATCGGCCTCTTCTTCCT	AGGACCACCAATCGACAGAC	qPCR

Supplementary Table 2: List of primary and secondary antibodies used in this study.

Antibody/ isotype	Company	Catalogue Number	Host Species	Dilution	Applications
**Primary Antibodies**					
Calbindin	Swant	CB38	Rabbit	1:5,000	IF/IHC/WB
IgG 647	Cell Signalling	2985S	Rabbit	1:300	FC
IgM 488	Biolegend	401617	Mouse	1:250	FC
Lamp2[H4B4]	Abcam	ab25631	Mouse	1:100/ 1:500	IF/ WB
LC3	Nanotool	0231-100/LC3-5F10	Mouse	1:200	IF
LC3B	Novus biologicals	NB100-2220	Rabbit	1:5,000	WB
NANOG 647	Cell Signalling	5448S	Rabbit	1:150	FC
Phospho-(Ser) PKC substrate	Cell Signalling	2261	Rabbit	1:1,000	WB
PKCγ (C19)	Santa Cruz	sc-211	Rabbit	1:100/1:400/1:500	IF/IHC/WB
PKCγ (C4)	Santa Cruz	sc-166385	Mouse	1:50	IF
pT514-PKCγ	Abcam	ab109539	Rabbit	1:1,000	WB
pT674-PKCγ	Abcam	ab5797	Rabbit	1:500	WB
Sodium potassium ATPase	Abcam	ab76020	Rabbit	1:100	IF
Tra-1-60 488	Biolegend	B119983	Mouse	1:100	FC
Ubiquitin	Abcam	ab7780	Rabbit	1:500	IF
β-actin	Abcam	ab3280	Mouse	1:1,000	WB
**Secondary Antibodies**					
anti-Goat Alexa 594	Invitrogen	A-11058	Donkey	1:1,000	IF
anti-Mouse Alexa 488	Invitrogen	A-11001	Goat	1:1,000	IF
anti-Mouse Alexa 594	Invitrogen	R37121	Goat	1:1,000	IF
anti-Rabbit Alexa 488	Invitrogen	R37116	Goat	1:1,000	IF
anti-Rabbit Alexa 594	Invitrogen	A-11012	Goat	1:1,000	IF
anti-Mouse HRP	GE Healthcare	NA931-1ML	Sheep	1:10,000	WB
anti-Rabbit HRP	GE Healthcare	NA934-1ML	Donkey	1:10,000	WB

FC: flow cytometry; IF: immunofluorescence; IHC: immunohistochemistry; WB: Western blotting

References

Nishimura K, Sano M, Ohtaka M, Furuta B, Umemura Y, Nakajima Y, et al. Development of defective and persistent Sendai virus vector: a unique gene delivery/expression system ideal for cell reprogramming. J Biol Chem 2011; 286: 4760-71. doi: 10.1074/jbc.m110.183780

Nishimura T, Kaneko S, Kawana-Tachikawa A, Tajima Y, Goto H, Zhu D, et al. Generation of rejuvenated antigen-specific T cells by reprogramming to pluripotency and redifferentiation. Cell Stem Cell 2013; 12: 114-26. doi: 10.1016/j.stem2012.11.002

Watson LM, Wong MMK, Vowles J, Cowley SA, Becker EBE. A Simplified Method for Generating Purkinje Cells from Human-Induced Pluripotent Stem Cells. Cerebellum Cerebellum 17:419–427. doi: 10.1007/s12311-017-0913-2
